# CASCC: a co-expression-assisted single-cell RNA-seq data clustering method

**DOI:** 10.1093/bioinformatics/btae283

**Published:** 2024-04-25

**Authors:** Lingyi Cai, Dimitris Anastassiou

**Affiliations:** Department of Systems Biology, Columbia University, New York, NY 10032, United States; Department of Electrical Engineering, Columbia University, New York, NY 10027, United States; Department of Systems Biology, Columbia University, New York, NY 10032, United States; Department of Electrical Engineering, Columbia University, New York, NY 10027, United States; Irving Comprehensive Cancer Center, Columbia University, New York, NY 10032, United States

## Abstract

**Summary:**

Existing clustering methods for characterizing cell populations from single-cell RNA sequencing are constrained by several limitations stemming from the fact that clusters often cannot be homogeneous, particularly for transitioning populations. On the other hand, dominant cell populations within samples can be identified independently by their strong gene co-expression signatures using methods unrelated to partitioning. Here, we introduce a clustering method, CASCC (co-expression-assisted single-cell clustering), designed to improve biological accuracy using gene co-expression features identified using an unsupervised adaptive attractor algorithm. CASCC outperformed other methods as evidenced by multiple evaluation metrics, and our results suggest that CASCC can improve the analysis of single-cell transcriptomics, enabling potential new discoveries related to underlying biological mechanisms.

**Availability and implementation:**

The CASCC R package is publicly available at https://github.com/LingyiC/CASCC and https://zenodo.org/doi/10.5281/zenodo.10648327.

## 1 Introduction

Single-cell RNA-sequencing (scRNA-seq) technologies have allowed the study of cellular heterogeneity and dynamics by providing transcriptome data at the level of individual cells. Specialized tools analyzing scRNA-seq data have been introduced, such as the following most widely used methods, CIDR (Lin *et al.* 2017), RaceID (Herman *et al.* 2018), SC3 (Kiselev *et al.* 2017), Seurat (Hao *et al.* 2021), SIMLR (Wang *et al.* 2017), and TSCAN (Ji and Ji 2016). An important methodology used in such tools is clustering into groups of cells with similar gene expression profiles, providing valuable insight into the presence of various cell types and subtypes as well as underlying biological mechanisms.

Existing clustering methods have limitations (Kiselev *et al.* 2019, Yu *et al.* 2022) as they attempt to partition complex sets of cells, often undergoing transition, into mutually exclusive subpopulations representing cell types. The results may only represent an approximation of biological reality. Clusters of cell types may merge or separate depending on parameter choices, so that their number and their separating borders may be arbitrary. Careful scrutiny of clustering results is important to avoid misleading conclusions (Kiselev *et al.* 2019) and determining whether clusters represent truly distinct populations is challenging.

Each cell is originally represented by its full gene expression profile vector. Using feature selection and dimensionality reduction effectively reduces the noise and accelerates the computational processes. Rather than each gene having equal contribution, the highly needed biological accuracy of the clustering results can be improved by emphasizing the features that are most relevant for the underlying heterogeneity and deemphasizing or removing the rest. Dimensionality reduction methods, like PCA helps project data into a lower-dimensional space for downstream analysis (Chari and Pachter 2023, Kiselev *et al.* 2019). To visualize the data, the dimensionality is further reduced to two dimensions (2D) using techniques such as t-SNE and UMAP, so that each cell can be depicted as a point in a plane.

Cell populations can be identified by their strong gene co-expression signatures using methods unrelated to partitioning, such as the iterative attractor algorithm (Cheng *et al.* 2013). Briefly, the algorithm starts from a “seed” gene and converges to an “attractor” gene signature. Each gene co-expression signature is uniquely identified by any choice of a seed gene belonging to that co-expression, and the algorithm is designed to converge to a ranked list of genes, identifying the genes at the core of co-expression. Such genes are particularly relevant to be used for feature selection in the clustering method.

Here, we introduce a co-expression-assisted single-cell clustering (CASCC) method for scRNA-seq analysis. A key feature of CASCC is the use of a novel adaptive attractor algorithm, which helps towards proper feature selection, clustering, and cell type identification, thus improving biological accuracy compared to existing methods.

## 2 Materials and methods

### 2.1 Algorithm description

**Figure 1.**
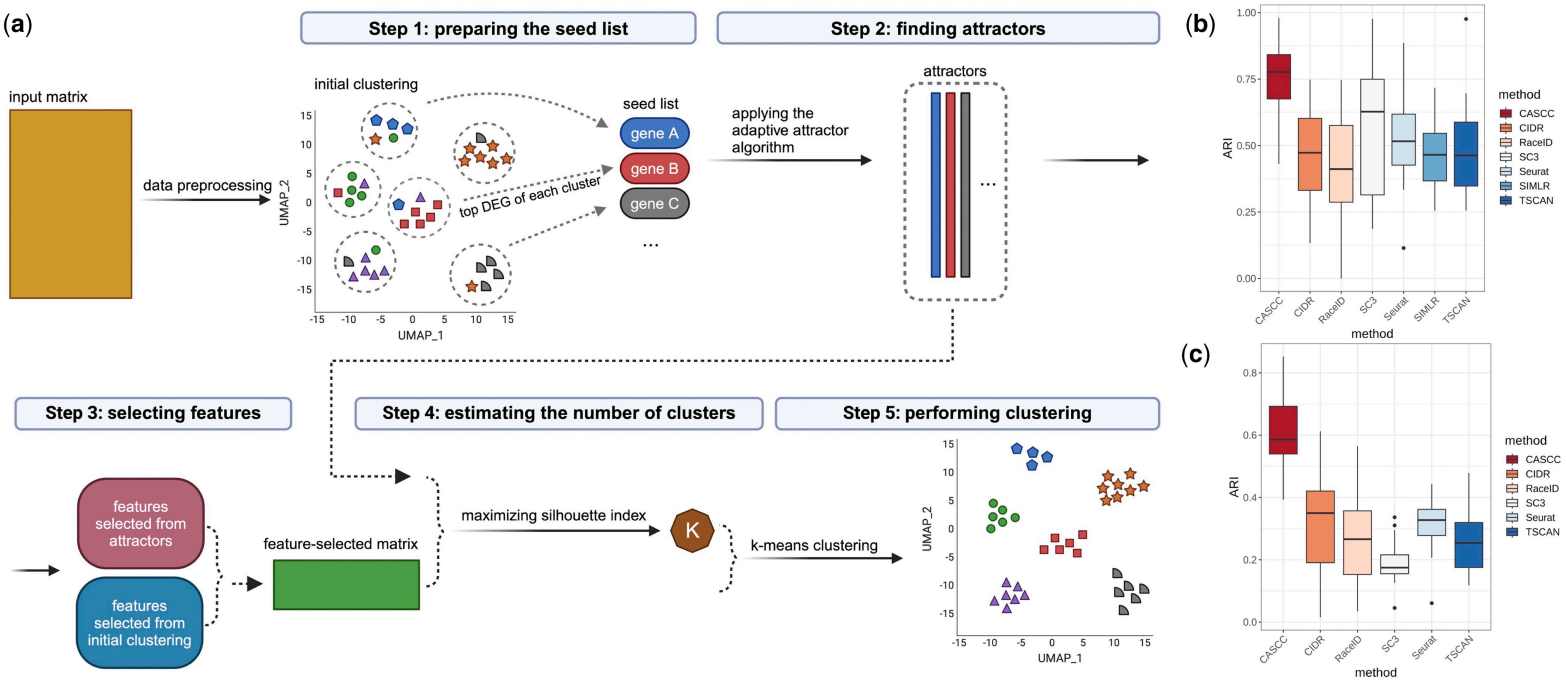
Overview and clustering performance of CASCC compared to other methods. (a) Overview of clustering with CASCC. Created with BioRender.com. (b) ARI scores of different clustering methods applied to 15 real datasets listed in Supplementary Table S1. The boxplot represents the interquartile range of the ARI, with the middle line indicating the median ARI among datasets. (c) ARI scores of six methods applied to 19 large-scale Tabula sapiens datasets.

A detailed description of CASCC including methods and evaluation metrics can be found in the Supplementary Material. Briefly, the CASCC algorithm comprises five main steps (Fig. 1a). We use an improved adaptive attractor algorithm that can be applied to scRNA-seq datasets. The algorithm iteratively finds co-expression signatures where the top-ranked genes are the most representative features. Each signature is defined by a list of ranked genes. Following an initial low computational complexity graph-based clustering (Hao *et al.* 2021), the top-ranked differentially expressed genes (DEGs) of each cluster are selected as features and as potential seeds used for the adaptive attractor method in the second step. The top co-expressed genes of each resulting attractor are also selected as features in the third step. The final number of clusters, K, is determined based on the attractor output in the fourth step. Lastly, K-means clustering is performed on the feature-selected expression matrix, in which the cells with the highest expression levels of attractors are chosen as the initial cluster centers.

### 2.2 Performance assessment

To ensure relevance and objectivity, we selected all 15 real scRNA-seq evaluation datasets suggested in a clustering benchmarking study (Krzak *et al.* 2019) representing various tissues and experimental protocols (Supplementary Table S1), which have been extensively used in previous studies to evaluate clustering performance. Each of them has no more than 3500 cells per dataset. So that we also evaluate our method on large-scale datasets, we selected the 19 datasets from different human organs from the Tabula Sapiens atlas (The Tabula Sapiens Consortium 2022) (Supplementary Table S2), each of which has at least 5000 and as many as 30 000 cells per dataset.

To assess and compare the accuracy of clustering by different techniques, we used the widely accepted metric of Adjusted Rand Index (ARI) (Hubert and Arabie 1985), but also other ones including Adjusted Mutual Information (AMI) and Normalized Mutual Information (NMI). We also used the metrics of the absolute log-modulus (Tran *et al.* 2022) and the deviation score (Yu *et al.* 2022) to test the accuracy of estimating the number of cell types, and the average silhouette width (ASW) (Rousseeuw 1987) to assess the 2D embeddings generated by different methods (Supplementary Material).

## 3 Results

We compared the clustering performance of CASCC against six most widely used methods for scRNA-seq clustering, including CIDR (Lin *et al.* 2017), RaceID3 (Herman *et al.* 2018), SC3 (Kiselev *et al.* 2017), Seurat V4 (Hao *et al.* 2021), SIMLR (Wang *et al.* 2017), and TSCAN (Ji and Ji 2016) (Supplementary Material). Other clustering methods with a requirement for users to set the number of clusters were not benchmarked for that reason. We evaluated their clustering results using the ARI metric on the 15 evaluation datasets mentioned above. Figure 1b shows that there is an improvement of 34% on mean ARI scores compared to the second-best performer SC3 (details can be found in Methods, Supplementary Table S3). AMI and NMI results can be found in Supplementary Fig. S1, in which CASCC is also the top performer. Wilcoxon test results show that CASCC achieved statistical significance differences when compared with the other methods (Supplementary Table S4).

To evaluate the clustering performance of our method on the latest large-scale datasets, we selected 19 datasets from the Tabula Sapiens atlas, ranging in size from 5000 to 30 000 cells. SIMLR’s peak memory usage is prohibitively high for large-scale datasets (Yu *et al.* 2022). Consistently, when executing SIMLR on a computing cluster with 400 GB memory, we always encountered “out of memory” errors. Thus, we compared our method with the other five methods. The ARI results presented in Fig. 1c indicate that CASCC has again the best performance.

We also evaluated the seven methods by measuring their accuracy in estimating the number of cell types, using metrics to compare the generated number of clusters with the true number of cell types (Supplementary Material). We observed that SC3 tends to significantly overestimate this number in large datasets with the highest mean absolute deviation value (Supplementary Table S5), which can be confusing, as some clusters may appear to lack clear biological significance (Vandenbon and Diez 2020). CASCC has the smallest absolute log-modulus values and the smallest absolute deviation among the methods indicating a favorable performance (Supplementary Table S5).

Another crucial task for scRNA-seq analysis is the 2D reduction and following visualization. CASCC and Seurat achieved much higher ASW values than those of the other methods, indicating better separation and cohesion of clusters (Supplementary Fig. S2).

Low computational complexity is desirable but not a strict requirement in clustering, as long as it is not prohibitive. Improving biological accuracy is the most important consideration to avoid potential inaccurate or misleading conclusions. We have included a parallel computing option in CASCC that allows a reduction of computing time. Compared to several methods, CASCC shows a reasonable level of complexity (Supplementary Table S6 and Supplementary Note).

## 4 Discussion

Our results indicate that CASCC consistently achieves high performance in clustering tasks across a wide range of data types, resulting in multiple benefits for subsequent analyses and improving the accuracy of the identification and interpretation of cell types. It can be used robustly on a variety of scRNA-seq protocols. Unlike the standard workflow, in which cell type markers are identified by DEGs computed after clustering, CASCC provides an additional perspective from the top-ranked genes of the co-expression signatures. The current version has limitations in scaling to atlas-level analysis, such as datasets of more than one million cells. With the rapid development of scRNA-seq technology, the next research focus will be to extend the application of CASCC to such datasets.

## Supplementary Material

btae283_Supplementary_Data

## Data Availability

All datasets used in this paper are publicly available. The annotated dataset suggested in a clustering benchmark study (Krzak *et al.* 2019), used to evaluate performance, consists of 15 real scRNA-seq datasets (data information can be found in Supplementary Table S1). We downloaded SingleCellExperiment objects of these datasets from https://hemberg-lab.github.io/scRNA.seq.datasets/. The 19 datasets (Supplementary Table S2) from human organ objects from the Tabula Sapiens atlas (The Tabula Sapiens Consortium 2022) are available at https://figshare.com/articles/dataset/Tabula_Sapiens_release_1_0/14267219.
