# Supplementary material for: CASCC: a co-expression-assisted single-cell RNA-seq data clustering method
Source: Bioinformatics. 2024 Apr 25;40(5):btae283. doi: 10.1093/bioinformatics/btae283 (PMC11091742; doi:10.1093/bioinformatics/btae283)
Supplement: btae283_Supplementary_Data [file btae283_supplementary_data.pdf]

Supplementary Material for

CASCC: A co-expression assisted single-cell RNA-seq data clustering method

Lingyi Cai, Dimitris Anastassiou*

*Corresponding author. Email: d.anastassiou@columbia.edu

Methods

1. The CASCC Algorithm

Data Preprocessing: The input of the CASCC is a scRNA-seq expression matrix, in which the rows and the columns represent the genes and the cells, respectively. First, the genes that are not expressed in any cells are removed. Second, to remove genes not relevant for characterizing cell types, CASCC filters out mitochondrial genes, ribosomal genes, and spike-in genes from the analysis by default (Ranjan *et al*., 2021), and consistently we found that this filtering step does not affect the overall accuracy of the results (Supplementary Figure S3). If the input matrix is well-normalized and log-transformed using any other methods, then CASCC uses the matrix directly. Otherwise, if the input matrix is in RPKM, FPKM, or TPM-normalized count, then the matrix is log2-transformed with a pseudo-count of 1, i.e., log2(norm_count +1). If the input matrix is in UMI-count, we first perform library size normalization to scale each cell by dividing by the total expression and multiplying by a scale factor (10,000), then log2-transform the matrix using the '*NormalizeData*' function in the *Seurat* R package.

The CASCC algorithm comprises five main steps: In Step 1, we apply initial clustering on a data matrix to identify the potential attractor seed genes. In Step 2, we apply a novel adaptive attractor algorithm, described below, to identify lists of co-expressed genes using the seed genes found in Step 1. Step 3 performs feature selection by selecting the top-ranked genes for each attractor found in Step 2, as well as the top-ranked differentially expressed genes (DEGs) of each cluster found in Step 1. The selected features are included in a "feature-selected matrix," which replaces the original data matrix. In Step 4, we reduce the feature-selected matrix of Step 3 to 30 PCA dimensions, on which we estimate the optimal number of clusters K based on the attractor output. In Step 5, we find centers and apply K-means clustering to the feature-selected matrix.

Step 1. Preparing the seed list.

To generate the initial clusters, we apply the low-complexity "Seurat" workflow to the gene expression matrix. The default parameters are used, except for the resolution parameter in the *FindCluster* function, which is increased to 2.0 to allow for potentially large number of clusters. As noted in previous studies (Kiselev *et al.,* 2017; Kiselev *et al.,* 2019), in small datasets Seurat may not identify all clusters. Therefore, if the number of cells is less than 300, we apply the K-

means clustering algorithm with default value of $K = 10$ to generate the initial clusters for downstream analysis.

For each cluster, we then identify the DEGs using the *FindAllMarkers* function and we add its top-ranked gene by fold change to the seed list. One of the parameters of the function, "*min.diff.pct*," specifies the difference in the fraction of detection between two groups required for the testing of particular genes. To reduce the number of false positive DEGs, we set min.diff.pct to 0.5, so that genes with at least a 50% difference in detection rate between the two groups are tested for differential expression. If the average number of DEGs detected among clusters is less than 5, we remove this restriction and include all genes in the differential expression analysis.

We thus obtain a list of seeds that represent the most characteristic markers of each cluster. Users have the option to extend the list by adding their own genes of interest.

Step 2. Applying the adaptive attractor algorithm.

To find co-expression signatures in the dataset, we apply the adaptive attractor algorithm described below to the gene expression matrix, using the seed list found in Step 1. The original attractor finding algorithm (Cheng *et al*., 2013), implemented in the *cafr* R package, finds mutually associated genes from an expression matrix in an iterative manner, converging to an "attractor" consisting of a ranked list of genes, based on a "weight" ranging from 0 to 1 assigned to each gene. Detailed descriptions can be found in (Cheng *et al*., 2013; Zhu *et al*., 2021). One of the parameters in the method is the exponent "a", determining the sharpness of co-expression. The most appropriate value of the parameter "a" depends on the specific characteristics of the datasets.

To optimize the algorithm, the adaptive attractor algorithm identifies an exponent maximizing the "strength" of the attractor, defined as the weight of the tenth gene, so that a sufficiently high number of co-expressed genes is included. It uses a "start exponent value" a_{max} (default = 10), an "end exponent value" a_{min} (default = 2), an "initial scanning step" $S_{initial}$ (default = 1), and a "small scanning step" S_{small} (default = 0.1). Starting from using the highest exponent (a_{max}), the attractor algorithm is applied to the data matrix for each seed obtained in Step 1, and the exponent is decreased stepwise aimed at maximizing the strength. If the strength stops increasing, or the seed gene drops out of the top *N* genes (default *N* = min(50, 0.5% × the total

number of genes)), which indicates divergence, then the exponent is increased by $S_{initial}$ and the scanning step is changed to S_{small}. The exponent is gradually decreased until the optimal (highest strength) attractor is found.

After obtaining a converged attractor, the ten top-ranked co-expressed genes and the genes with rankings higher than the seed gene in that attractor are removed from the list of remaining seeds, as they are unlikely to converge to a different attractor if used as seeds. The top-ranked genes in each attractor are co-expressed and likely to represent a cell type. Let *M* be the number of found attractors after scanning all genes in the seed list and removing the identical attractors.

Step 3. Selecting features.

The set of selected features includes the union of the top 50 genes of each attractor and the top 10 DEGs in each cluster. This choice of default values for the number of selected features was made as we found that the average performance would not improve using higher numbers for those features, resulting in a feature-selected matrix with a reduced number of rows.

Step 4. Estimating the number of clusters.

We reduce the feature-selected matrix of Step 3 to 30 PCA dimensions to preserve the global structure (Chari and Pachter 2023). This dimensional reduction parameter has been incorporated in CASCC R package, allowing users the flexibility to modify the number of dimensions according to their needs. To determine the proper number of clusters, we apply to the resulting PCA embeddings the *NbClust* function from the *NbClust* R package, maximizing the "silhouette index" (Rousseeuw 1987). Two parameters of the *NbClust* function, *min.nc* and *max.nc*, determine the minimal and maximal numbers of clusters, respectively.

The value of *min.nc* is defined as follows. We identify the number *N* of attractors with strong co-expression according to the criteria used in a previous study (Zhu and Anastassiou 2020, algorithm step 2 in the supplementary material): Among the *M* attractors found in Step 2, we first eliminate any attractor for which the weight difference between the top two genes is greater than 0.2, suggesting that the top gene is not part of uniform co-expression. Second, we only keep attractors in which the weight of the 5th-ranked gene is at least 0.4, which indicates that at least five genes are involved in a sufficiently strong co-expression mechanism. We then set *min.nc* = max(3, *N*).

The value of *max.nc* is defined as the number of attractors found in Step 2 after removing “duplicate” attractors, defined as those for which there is overlap in at least *overlapN* (default = 10, sensitivity analysis of parameter *overlapN* can be found in Supplementary Note 2) of the top 50 genes, likely to represent the same population, filtering out those with largest difference between the top two genes.

The number of clusters, *K*, is then determined by the function *NbClust* using the above parameters and is used for the final Step 5. Users can also manually set *K* according to their prior knowledge.

Step 5. Performing clustering.

We use the K-means algorithm with specified centers to cluster cells. To find appropriate centers, the cells with the highest expression levels of the average of the top five genes of each among those M attractors are chosen as the initial cluster centers for the K-means algorithm. If $M > K$, by selecting the K attractors with the smallest difference of weights between the top two genes. We use the ‘*kmeans*’ function in the *stats* R package with the default ‘Hartigan-Wong’ algorithm.

2. Figures of merit

2.1 ARI, AMI and NMI

We used three evaluation metrics detailed below to assess the quality of clustering results by comparing them to the ground truth.

Given a set of *N* cells, let *X* and *Y* be two sets of clustering labels (vectors of length *N*) for these cells. To measure the agreements between *X* and *Y*, we calculated the Adjusted Rand Index (ARI), Adjusted Mutual Information (AMI), and Normalized Mutual Information (NMI) implemented in the *aricode* R package (version 1.0.0) (Chiquet *et al.*, 2020).

The ARI is defined as follows (Hubert and Arabie 1985):

$$ARI = \frac{\sum_{ij}\binom{n_{ij}}{2} - \left[\sum_i\binom{a_i}{2}\sum_j\binom{b_j}{2}\right]\Big/\binom{N}{2}}{\frac{1}{2}\left[\sum_i\binom{a_i}{2} + \sum_j\binom{b_j}{2}\right] - \left[\sum_i\binom{a_i}{2}\sum_j\binom{b_j}{2}\right]\Big/\binom{N}{2}}$$

The variables n_{ij}, a_i and b_j are derived from the contingency table, which is a matrix with elements n_{ij} denoting the overlap between the two partitions. The number of rows of the contingency table is equal to the number of clusters in X, while the number of columns is equal to the number of clusters in Y. The value of n_{ij} is the number of times a cell appears in both cluster i of X and cluster j of Y. The values of a_i and b_j are the sums of the elements in the ith row and the jth column, respectively. The value of ARI varies from -1 to 1, so that ARI = 1 implies that the two clustering results are matching perfectly.

The AMI (Vinh *et al.*, 2009) is defined as:

$$AMI = \frac{I(X,Y) - E\{I(X,Y)\}}{max\{H(X),H(Y)\} - E\{I(X,Y)\}}$$

$I(X,Y)$ refers to the mutual information between X and Y, while $H(X)$ and $H(Y)$ represent entropies. $E\{I(X,Y)\}$ is the expected mutual information between X and Y. The value of AMI varies from -1 to 1.

The NMI (Strehl and Ghosh 2003) is defined as follows and its value varies from 0 to 1:

$$NMI = \frac{I(X,Y)}{max\{H(X),H(Y)\}}$$

2.2 ASW

The Average Silhouette Width (ASW) is a well-established criterion to quantify the closeness of cells within the same cluster, compared to their separation from cells in different clusters (Rousseeuw 1987; Ranjan *et al.,* 2021). The ASW score falls within the range of -1 to 1, with a high score indicating better results. To evaluate the ASW, we use the *silhouette* function in the *cluster* R package (version 2.1.3) and the *SilhouetteBenchmarkPerCTPCA* function (Ranjan *et al.,* 2021) on the 2-D reduction output produced by different clustering methods.

2.3 Absolute log-modulus

We evaluate the ability of different methods to estimate the number of cell types using the absolute log-modulus metric (John and Draper 1980; Tran *et al.,* 2022):

$$L(x) = |sign(x) \times log_{10}(|x| + 1)|$$

in which x represents the difference between the actual and estimated number of cell types. A

lower value of $L(x)$ value indicates higher accuracy.

2.4 Deviation

To evaluate overestimation or underestimation in cell type estimation, we use the metric adopted by the benchmarking paper (Yu *et al.*, 2022):

$$deviation = (\#predicted\_cell\_types - \#true\_cell\_types) / \#true\_cell\_types$$

in which a positive deviation value represents overestimation, while a negative deviation value represents underestimation.

3. Availability of data and software packages

The R package of CASCC is publicly available at GitHub: https://github.com/LingyiC/CASCC. The submitted software version is also archived at the Zenodo repository: https://zenodo.org/doi/10.5281/zenodo.10648327. The practical use of CASCC is described in Supplementary Note 3.

All datasets used in this paper are publicly available.

The annotated dataset suggested in a clustering benchmark study (Krzak *et al.,* 2019), used to evaluate performance, consists of 15 real scRNA-seq datasets. They have cells that vary from 50 cells to 3,500 cells, genes that vary from 15,000 genes to 55,000 genes (data information can be found in Supplementary Table S1). We downloaded SingleCellExperiment objects of these datasets from https://hemberg-lab.github.io/scRNA.seq.datasets/.

The 19 datasets (Supplementary Table S2) ranging in size from 5,000 to 30,000 cells from human organ objects from the Tabula Sapiens atlas (The Tabula Sapiens Consortium, 2022) are available at https://figshare.com/articles/dataset/Tabula_Sapiens_release_1_0/14267219.

All clustering methods used in the evaluation are available in R packages. The specific package version used in this study is indicated as follows: Seurat (v4.3.0), CIDR (v0.1.5), RaceID (v0.3.0), SC3 (v1.18.0), SIMLR (v1.16.0), TSCAN (v2.0.0).

Supplementary Tables

Table S1. Krzak *et al* datasets information. Detailed information can be found in table 1 and table 2 in (Krzak *et al.,* 2019) and studies listed in the "Reference" column.

	Dataname	#Gene	#Cell	Protocol	Dataset	Reference
**1**	Brain_darmanis	22088	466	SMARTer	GSE67835	Darmanis *et al.*, 2015
**2**	Brian_romanov	24341	2881	Fluidigm C1	GSE74672	Romanov *et al.*, 2017
**3**	Brian_tasic	24057	1679	SMARTer	GSE71585	Tasic *et al.*, 2016
**4**	Brian_zeisel	19972	3005	STRT/C1 UMI	GSE60361	Zeisel *et al.*, 2015
**5**	Embryo_Devel_biase	25734	56	SMARTer	GSE57249	Biase *et al.*, 2014
**6**	Embryo_Devel_deng	22431	268	Smart-seq	GSE45719	Deng *et al.*, 2014
**7**	Embryo_Devel_goolam	41428	124	Smart-seq2	E-MTAB-3321	Goolam *et al.*, 2016
**8**	Embryo_Devel_yan	20214	90	Tang	GSE36552	Yan *et al.*, 2013
**9**	Embryo_Stem_klein	24175	2717	inDrop	GSE65525	Klein *et al.*, 2015
**10**	Embryo_Stem_kolodziejczyk	38616	704	SMARTer	E-MTAB-2600	Kolodziejczyk *et al.*, 2015
**11**	Pancreas_baron-mouse	14878	1886	inDrop	GSE84133	Baron *et al.*, 2016
**12**	Pancreas_segerstolpe	25525	3514	Smart-seq2	E-MTAB-5061	Segerstolpe *et al.*, 2016
**13**	Pancreas_xin	39851	1600	SMARTer	GSE81608	Xin *et al.*, 2016
**14**	Tissues_li	55186	561	SMARTer	GSE81861	Li *et al.*, 2017
**15**	Tissues_treutlein	23271	80	SMARTer	GSE52583	Treutlein *et al.*, 2014

Table S2. Tabula Sapiens datasets information. Description of large-scale Tabula Sapiens datasets from 19 organs used in this study.

	Dataname	#Gene	#Cell	Protocol		Dataname	#Gene	#Cell	Protocol
**1**	TS_Bladder	58870	24583	10x, smart-seq2	**11**	TS_Pancreas	58870	13497	10x, smart-seq2
**2**	TS_Bone_Marrow	58870	12297	10x, smart-seq2	**12**	TS_Prostate	58870	16375	10x, smart-seq2
**3**	TS_Eye	58870	10650	10x, smart-seq2	**13**	TS_Salivary_Gland	58870	27199	10x, smart-seq2
**4**	TS_Fat	58870	20263	10x, smart-seq2	**14**	TS_Skin	58870	9424	10x, smart-seq2
**5**	TS_Heart	58870	11505	10x, smart-seq2	**15**	TS_Small_Intestine	58870	12467	10x, smart-seq2
**6**	TS_Kidney	58870	9641	10x, smart-seq2	**16**	TS_Tongue	58870	15020	10x, smart-seq2
**7**	TS_Large_Intestine	58870	13680	10x, smart-seq2	**17**	TS_Trachea	58870	9522	10x, smart-seq2
**8**	TS_Liver	58870	5007	10x, smart-seq2	**18**	TS_Uterus	58870	7124	10x, smart-seq2
**9**	TS_Mammary	58870	11375	10x, smart-seq2	**19**	TS_Vasculature	58870	16037	10x, smart-seq2
**10**	TS_Muscle	58870	30746	10x, smart-seq2					

Table S3. ARI results for Krzak *et al* datasets. The highest ARI score of each dataset is in red font.

	CASCC	CIDR	RaceID	SC3	Seurat	SIMLR	TSCAN
**Brain_darmanis**	**0.7053**	0.4578	0.0624	0.6135	0.5503	0.5428	0.5627
**Brain_romanov**	**0.7374**	0.3015	0.4199	0.1873	0.3339	0.3771	0.3644
**Brain_tasic**	0.6457	0.2675	0.3092	**0.8525**	0.6987	0.4841	0.3314
**Brain_zeisel**	**0.8970**	0.3617	0.7062	0.3604	0.4711	0.7177	0.3672
**Embryo_Devel_biase**	0.6388	0.6076	0.7464	0.8698	0.5814	0.4647	**0.9760**
**Embryo_Devel_deng**	0.4310	**0.7483**	0.4116	0.5549	0.4592	0.2550	0.5825
**Embryo_Devel_goolam**	**0.9808**	0.5972	0.2135	0.6299	0.5821	0.4425	0.6408
**Embryo_Devel_yan**	**0.8014**	0.5945	0.6390	0.6276	0.6911	0.4654	0.4630
**Embryo_Stem_klein**	**0.8434**	0.7113	0.3176	0.6368	0.6546	0.5611	0.6965
**Embryo_Stem_kolodziejczyk**	**0.8766**	0.4730	0.2648	0.8296	0.4461	0.3575	0.4927
**Pancreas_baron-mouse**	**0.8409**	0.4617	0.4251	0.2683	0.5162	0.3417	0.2616
**Pancreas_segerstolpe**	**0.6216**	0.2610	-0.0002	0.1910	0.3852	0.2952	0.3182
**Pancreas_xin**	**0.7978**	0.5854	0.3789	0.2273	0.4067	0.5494	0.4440
**Tissues_li**	0.7505	0.6826	0.5125	**0.9768**	0.8857	0.6778	0.5942
**Tissues_treutlein**	**0.7770**	0.1327	0.6698	0.6698	0.1146	0.4996	0.2559

Table S4. Wilcoxon test comparing CASCC and other methods. The significance of differences between six clustering methods and CASCC, measured by three metrics among datasets in Table S1. Asterisks (*) indicate p-value < 0.05.

method	ARI		AMI		NMI	
**CIDR**	1.16E-03	*	6.10E-05	*	6.10E-05	*
**RaceID**	3.05E-04	*	3.05E-04	*	1.83E-04	*
**SC3**	7.30E-02		4.13E-02	*	4.13E-02	*
**Seurat**	1.53E-03	*	2.01E-03	*	2.01E-03	*
**SIMLR**	6.10E-05	*	6.10E-05	*	6.10E-05	*
**TSCAN**	2.62E-03	*	6.71E-03	*	3.36E-03	*

Table S5. Accuracy in estimating the number of cell types. The columns show the average absolute log-modulus and the average absolute deviation among datasets in Table S1. The best performance of each metric is in red font.

	Mean absolute log modulus	Mean absolute deviation
**CASCC**	**0.4108**	**0.2860**
**CIDR**	0.6365	0.5954
**RaceID**	0.5817	0.6791
**SC3**	0.7475	1.0698
**Seurat**	0.5079	0.5665
**SIMLR**	0.5852	0.6698
**TSCAN**	0.4138	0.3410

Table S6. Computing time in minutes for different methods when applied to the Krzak *et al* datasets. The number of genes and number of cells are listed in Table S1. Across 15 datasets, the "Minimum" column shows the shortest elapsed time, while the "Maximum" column shows the longest elapsed time. Methods are sorted by the average running time (Supplementary Note 1).

	Minimum	Maximum	Average
**Seurat**	0.05	0.28	0.16
**TSCAN**	0.01	3.42	0.71
**CIDR**	0.01	6.34	1.39
**SC3***	0.44	12.38	3.95
**CASCC***	0.71	78.69	20.36
**SC3**	0.40	83.66	20.58
**SIMLR***	0.15	131.53	28.88
**SIMLR**	0.66	158.63	35.90
**RaceID***	0.04	459.66	71.20
**RaceID**	0.04	471.18	72.02
**CASCC**	1.44	415.17	113.15

Asterisks (*) indicate parallel computing

Supplementary Figures

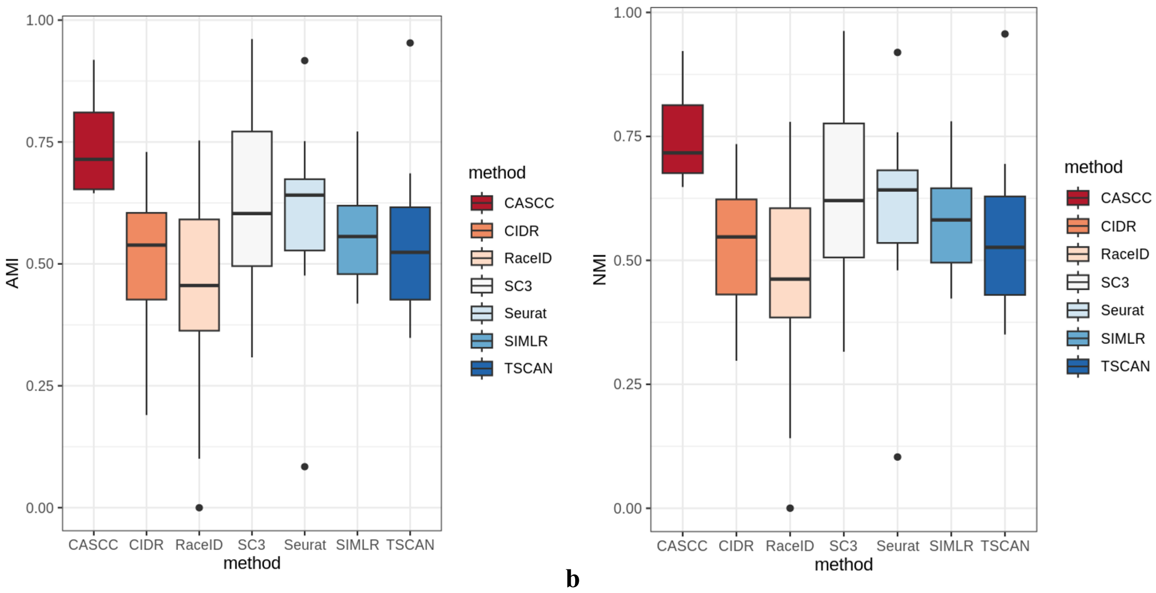

Figure S1. Benchmarking of clustering methods using AMI and NMI metrics. (a) AMI of seven clustering methods applied to the Krzak *et al* datasets in Table S1. The boxplot represents the interquartile range (IQR) of the AMI, with the middle line indicating the median AMI among datasets. **(b)** NMI of different clustering methods.

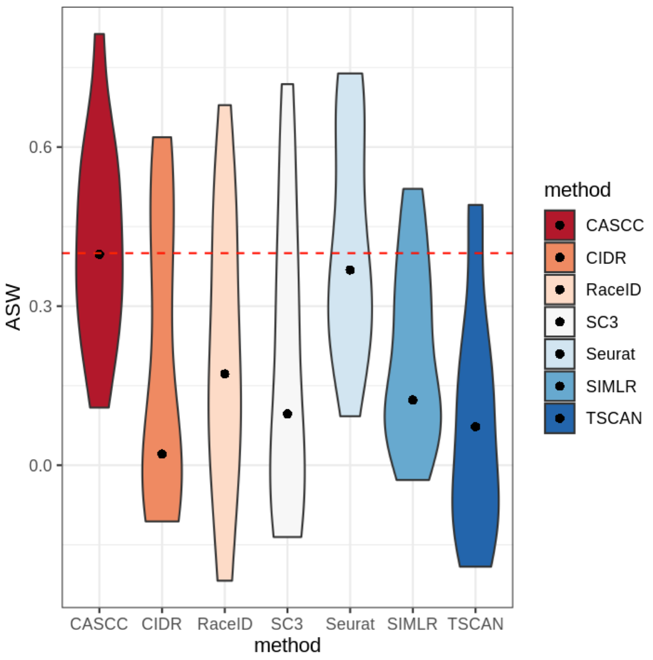

Figure S2. Comparison of the ASW across the Krzak *et al* datasets in Table S1. The median value is indicated by the black point.

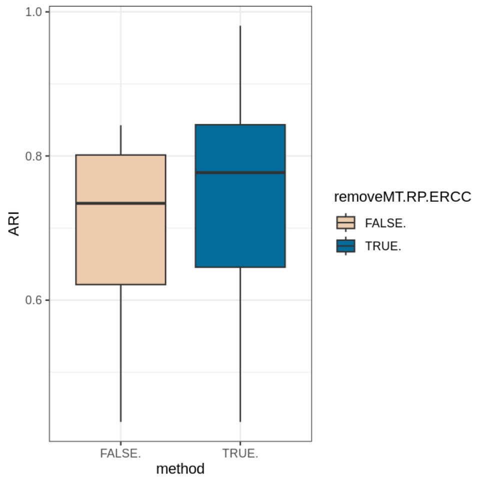

Figure S3. Comparison of ARIs with and without filtering gene steps. Comparison of ARI scores with and without filtering mitochondrial genes, ribosomal genes, and spike-in genes. Boxplots showing the distribution of clustering accuracy in the CASCC analysis pipeline with and without filtering those genes among the datasets in Table S1.

Supplementary Notes

1. Benchmarking computational time

We first benchmarked the computing time of all methods using a single CPU core. Seurat, TSCAN, and CIDR are the three fastest methods, with an average running time of less than two minutes (Supplementary Table S6). As the other four methods (CASCC, RaceID, SC3, SIMLR) with higher complexity can be run in parallel, we then benchmarked these methods with multi-cores as well (e.g., 20 CPU cores in the evaluation). CASCC shows a reasonable level of computation time with parallel computing enabled. Overall, we found that Seurat is the fastest method. We used the *sys.time* function implemented in R to evaluate computation efficiency. Computing time was evaluated on an Intel(R) Xeon(R) Gold 6226R CPU @ 2.90GHz.

2. The sensitivity analysis of parameter *overlapN*

Parameter *overlapN* is set to detect duplicated attractors, which is related to the estimation of the number of clusters. A higher value of *overlapN* tends to result in a greater number of clusters, as it sets a higher threshold for attractor removal (Methods/The CASCC Algorithm). We performed a sensitivity analysis of parameter *overlapN* under different choices evaluated by ARI (Methods/Figures of merit). The performance of CASCC shows strong robustness under different choices of the *overlapN* parameter ranging from 5 to 50, achieving a consistently high average ARI from 0.70 to 0.76, with only 9% change between the maximum and minimum average ARIs. We assign *overlapN* = 10 as the default parameter as it is one of the corresponding settings that achieves the highest average ARI score.

3. Practical use of CASCC

CASCC is a R package designed to perform clustering using gene co-expression features identified using an unsupervised adaptive attractor algorithm. This supplementary note provides a brief overview of how to use CASCC for clustering and attractor signatures identification.

Applying CASCC for clustering and attractor signature identification.

```r
rm(list = ls()); gc()
library(CASCC)

# clustering
## load the example dataset
data("Data_PDAC_peng_2k")
dim(Data_PDAC_peng_2k) # 400 cells
## run CASCC
res <- CASCC::run.CASCC(Data_PDAC_peng_2k)
## clustering results
labels <- res$mainType.output$clusteringResults

# finding attractor only
# Find LUM-seeded attractor. LUM gene is a marker of fibroblast cells.
attr.res <- findAttractor.adaptive(Data_PDAC_peng_2k, "LUM")
attr <- attr.res$attractor.final
```

References

Baron M, Veres A, Wolock SL *et al.,* A Single-Cell Transcriptomic Map of the Human and Mouse Pancreas Reveals Inter- and Intra-cell Population Structure. *Cell Syst.,* 2016;**3**:346-360.e4.

Biase FH, Cao X, Zhong S. Cell fate inclination within 2-cell and 4-cell mouse embryos revealed by single-cell RNA sequencing. *Genome Res.,* 2014;**24**:1787–96.

Chari T. and Pachter L. The specious art of single-cell genomics. *PLoS Comput. Biol.,* 2023; **19**: e1011288.

Cheng W-Y, Ou Yang T-H, Anastassiou D. Biomolecular Events in Cancer Revealed by Attractor Metagenes. *PLoS Comput. Biol.*, 2013;**9**:e1002920.

Chiquet J, Rigaill G, Sundqvist M. *Aricode: Efficient Computations of Standard Clustering Comparison Measures*., 2020.

Darmanis S, Sloan SA, Zhang Y *et al.,* A survey of human brain transcriptome diversity at the single cell level. *Proc. Natl. Acad. Sci.*, 2015;**112**:7285–90.

Deng Q, Ramsköld D, Reinius B *et al.,* Single-Cell RNA-Seq Reveals Dynamic, Random Monoallelic Gene Expression in Mammalian Cells. *Science*, 2014;**343**:193–6.

Goolam M, Scialdone A, Graham SJL *et al.,* Heterogeneity in Oct4 and Sox2 Targets Biases Cell Fate in 4-Cell Mouse Embryos. *Cell*, 2016;**165**:61–74.

Hubert L, Arabie P. Comparing partitions. *J Classif.*, 1985;**2**:193–218.

John JA, Draper NR. An Alternative Family of Transformations. *J R Stat Soc Ser C Appl. Stat.*, 1980;**29**:190–7.

Kiselev VY, Andrews TS, Hemberg M. Challenges in unsupervised clustering of single-cell RNA-seq data. *Nat. Rev. Genet.*, 2019;**20**:273–82.

Kiselev VY, Kirschner K, Schaub MT *et al.,* SC3: consensus clustering of single-cell RNA-seq data. *Nat. Methods*, 2017;**14**:483–6.

Klein AM, Mazutis L, Akartuna I *et al.,* Droplet Barcoding for Single-Cell Transcriptomics Applied to Embryonic Stem Cells. *Cell*, 2015;**161**:1187–201.

Kolodziejczyk AA, Kim JK, Tsang JCH *et al.,* Single Cell RNA-Sequencing of Pluripotent States Unlocks Modular Transcriptional Variation. *Cell Stem Cell*, 2015;**17**:471–85.

Krzak M, Raykov Y, Boukouvalas A *et al.,* Benchmark and Parameter Sensitivity Analysis of Single-Cell RNA Sequencing Clustering Methods. *Front. Genet.*, 2019;**10**.

Li H, Courtois ET, Sengupta D *et al.,* Reference component analysis of single-cell transcriptomes elucidates cellular heterogeneity in human colorectal tumors. *Nat. Genet.*, 2017;**49**:708–18.

Ranjan B, Sun W, Park J *et al.,* DUBStepR is a scalable correlation-based feature selection method for accurately clustering single-cell data. *Nat. Commun.*, 2021;**12**:5849.

Romanov RA, Zeisel A, Bakker J *et al.,* Molecular interrogation of hypothalamic organization reveals distinct dopamine neuronal subtypes. *Nat. Neurosci.*, 2017;**20**:176–88.

Rousseeuw PJ. Silhouettes: A graphical aid to the interpretation and validation of cluster analysis. *J. Comput. Appl. Math.*, 1987;**20**:53–65.

Segerstolpe Å, Palasantza A, Eliasson P *et al.,* Single-Cell Transcriptome Profiling of Human Pancreatic Islets in Health and Type 2 Diabetes. *Cell Metab.*, 2016;**24**:593–607.

Strehl A, Ghosh J. Cluster ensembles --- a knowledge reuse framework for combining multiple partitions. *J. Mach. Learn. Res.*, 2003;**3**:583–617.

Tasic B, Menon V, Nguyen TN *et al.,* Adult mouse cortical cell taxonomy revealed by single cell transcriptomics. *Nat. Neurosci.*, 2016;**19**:335–46.

The Tabula Sapiens Consortium. The Tabula Sapiens: A multiple-organ, single-cell transcriptomic atlas of humans. *Science*, 2022;**376**:eabl4896.

Tran B, Tran D, Nguyen H *et al.,* scCAN: single-cell clustering using autoencoder and network fusion. *Sci. Rep.*, 2022;**12**:10267.

Treutlein B, Brownfield DG, Wu AR *et al.,* Reconstructing lineage hierarchies of the distal lung epithelium using single-cell RNA-seq. *Nature*, 2014;**509**:371–5.

Vandenbon A, Diez D. A clustering-independent method for finding differentially expressed genes in single-cell transcriptome data. *Nat. Commun.*, 2020;**11**:4318.

Vinh NX, Epps J, Bailey J. Information theoretic measures for clusterings comparison: is a correction for chance necessary? *Proceedings of the 26th Annual International Conference on Machine Learning*., New York, NY, USA: Association for Computing Machinery, 2009, 1073–80.

Xin Y, Kim J, Okamoto H *et al.,* RNA Sequencing of Single Human Islet Cells Reveals Type 2 Diabetes Genes. *Cell Metab.*, 2016;**24**:608–15.

Yan L, Yang M, Guo H *et al.,* Single-cell RNA-Seq profiling of human preimplantation embryos and embryonic stem cells. *Nat. Struct. Mol. Biol.*, 2013;**20**:1131–9.

Yu L, Cao Y, Yang JYH et al., Benchmarking clustering algorithms on estimating the number of cell types from single-cell RNA-sequencing data. *Genome Biol.*, 2022;**23**:49.

Zeisel A, Muñoz-Manchado AB, Codeluppi S *et al.,* Cell types in the mouse cortex and hippocampus revealed by single-cell RNA-seq. *Science*, 2015;**347**:1138–42.

Zhu K, Anastassiou D. 2DImpute: imputation in single-cell RNA-seq data from correlations in two dimensions. *Bioinformatics*, 2020;**36**:3588–9.

Zhu K, Cai L, Cui C et al. Single-cell analysis reveals the pan-cancer invasiveness-associated transition of adipose-derived stromal cells into COL11A1-expressing cancer-associated fibroblasts. *PLoS Comput. Biol.*, 2021;**17**:e1009228.
